# Analysis of Sasang Constitutional Medicine as an Optimal Preventive Care Strategy for Hemophilia Patients

**DOI:** 10.1155/2020/4147803

**Published:** 2020-02-01

**Authors:** Mi Kyung Lee, Minwoo Hwang, Hyunjoo Oh, Kyoung Soo Kim

**Affiliations:** ^1^Sasang Medicine Clinic, Kyung Hee University Hospital at Gangdong, 892 Dongnam-ro, Gangdong-gu, Seoul 05278, Republic of Korea; ^2^Department of Sasang Constitutional Medicine, College of Korean Medicine, Kyung Hee University, 26 Kyungheedae-ro, Dongdaemun-gu, Seoul 02447, Republic of Korea; ^3^Department of Clinical Korean Medicine, College of Korean Medicine, Graduate School, Kyung Hee University, Seoul, Republic of Korea; ^4^Department of Clinical Pharmacology and Therapeutics, Kyung Hee University School of Medicine, 26 Kyungheedae-ro, Dongdaemun-gu, Seoul 02447, Republic of Korea

## Abstract

*Introduction*. Medical improvements have allowed hemophilia patients to anticipate an increased quality of life and life expectancy similar to that of the general population. Analysis of the potential disease symptoms of hemophilia patients based on a survey of Sasang Constitutional Medicine (SCM) is important for optimal preventive care and adjunctive therapy to avoid life-threating complications.

**Aim:**

To predict potential disease symptoms from the viewpoint of SCM as a preventive care strategy for hemophilia patients.

**Methods:**

Sixty-one hemophilia patients responded to a survey on Sasang constitutional classification, hemophilia disease pattern, and original symptoms.

**Results:**

In terms of SCM type, the 61 of hemophilia patients included 37 Tae-Eum (60.7%), 18 So-Yang (29.5%), and 6 So-Eum (12.5%). Hemophilia was found to be higher in Tae-Eum type and lower in So-Yang and So-Eum types, while considering the distributional rate of Korean Sasang types. Most of the patients with Tae-Eum type had Joyeol or Ganyeol. Furthermore, the incidences of diabetes and high blood pressure were greater in Tae-Eum type than in those of other types.

**Conclusion:**

In order to increase the quality of life and overall life expectancy, hemophilia patients with Tae-Eum type should be treated through management according to SCM along with medicine against hemophilia as long-term preventive care. Diabetes and high blood pressure should be regularly monitored in patients with Tae-Eum type.

## 1. Introduction

Hemophilia is the most common X-linked genetic disorder and is characterized by improper blood clotting [1]. This can lead to bleeding after injury or surgery, as well as spontaneous bleeding. Blood contains a series of proteins called coagulation protein factors that help to prevent bleeding. Hemophilia type A and B patients are mainly deficient in factors VIII and IV, respectively, which are essential for coagulation. These deficiencies can lead to defects in coagulation and cause spontaneous bleeding, affecting soft tissues, joints, and muscles [2].

As a hemophilic child grows to adolescence, bleeding occurs more frequently in the joints and muscles. Repeated joint or intramuscular hemorrhaging can lead to hemophilic arthritis, resulting in severe dysfunctions that can profoundly affect daily life, such as joint pain, joint deformation, and limited range of motion [3]. Hemorrhagic prophylaxis, routine home health care, and intravenous infusion are initiated during childhood. In addition to medical treatment for a lifetime, self-management and preventive care are important for the patient's quality of life. Thus, it is thought that Sasang Constitutional Medicine (SCM), a traditional Korean form of personalized medicine, will enhance patients' quality of life as a method of preventive care and adjunctive therapy [4].

Sasang Constitutional Medicine (SCM) was invented by Lee Je-ma and described in his book “The Principal of Life Preservation in Oriental Medicine” (Dongyisusebowon) [5]. In this book, the author explains that people can be divided into four types using their psychological, physical, and genetic characteristics; thus, even the same disease should be treated and prevented differently according to the individual's Sasang type. Sasang medicine was a theory based on long-term clinical experience of the author and the traditional classical literature on type. In Sasang Constitutional Medicine, people are divided into four types: So-Yang, Tae-Eum, So-Eum, and Tae-Yang. The temperament and character profiles of adults according to Sasang typology have been well described [6]. For instance, the So-Yang type is associated with a sharp and clean-looking person who is extroverted, hot-tempered, and interested in the outside world. The So-Eum type is described as an inactive, prudent, narrow-minded, negative, organized, nervous, and resolute person. The Tae-Yang type is characterized as a creative, good-headed, judgmental, and enterprising person. The Tae-Eum type is calm and cheerful, has a diligent personality, eats a lot, and is dedicated. This classification is also based on a comprehensive understanding of a person's physique, facial appearance, personality, emotions, and reactions to medications [7, 8]. The causes of illnesses and susceptibilities to disorders vary according to Sasang type [9–11]. There are also type-specific pathophysiological symptoms (TSPS), and the reactivity of medical herbs differs according to the Sasang type [12, 13]. Furthermore, SCM can help to explain the genetic differences among individuals [14, 15]. Therefore, the information from each individual's Sasang typology is very helpful for diagnosing and treating patients on the basis of combined treatments. In this study, for the first time, we investigated Sasang typology as an optimal preventive care strategy for hemophilia patients.

## 2. Materials and Methods

### 2.1. Patients

Sixty-one hemophilia patients who participated in a hemophilia welfare camp responded to a survey on Sasang constitutional classification, hemophilia disease pattern, and original symptoms (Table 1). All the questionnaires included the four Sasang constitution types and the pattern and degree of disease.

### 2.2. Classification of Patients by Sasang Typology

From August 2015 to August 2017, 61 hemophiliacs who participated in a hemophilia welfare camp were asked to complete three questionnaires regarding their hemophilia diagnosis, Sasang typology, and So-Jeung (original symptoms) diagnosis. Facial images (frontal and lateral) were taken. On the basis of the facial images and the three questionnaires, patients were classified into one of four Sasang types. Because Tae-Yang is a very rare Sasang type, the participants were classified into one of the three traditional Korean Sasang types (So-Yang, Tae-Eum, So-Eum) by three specialists in Sasang typology.

### 2.3. Questionnaire for Sasang Constitution Classification

Two questionnaires were used to classify patients according to Sasang typology as described below.

#### 2.3.1. Questionnaire for Sasang Constitution Classification II (QSCC2+)

QCSS2 is a questionnaire of PC program for the objective diagnosis of Sasang Constitution Classification [16]. This test was standardized from several existing questionnaires, is highly accurate, and can be validated through physical examination. Thus, it can provide diagnostic indicators. It consists of 121 questions, 15 of which are multiple-choice questions, and the rest of which are subjective. There are 80 questions about nature and emotion.

#### 2.3.2. Questionnaire for So-Jeung (Original Symptoms)

So-Jeung (original symptoms) are symptoms that usually appear according to an individual's Sasang constitution. These symptoms can be observed when an imperfect state of the body's metabolic function continues to deteriorate without proper treatment. Such symptoms will develop into future disease. They are diagnosed according to the characteristics of sleep, digestion, bowel movements, urine, sweat, and heat sensitivity. The definition of the original symptoms was reviewed in a previous study and has been a key concept addressing the innate symptoms of each type of constitution [5].

### 2.4. Analysis of Facial Images for Sasang Typology

More exact analysis of Sasang typology requires facial images for the analysis of features such as head length, breadth, and circumference; face length; sagittal arc; and bitragion arc [17].

### 2.5. Statistical Analysis

Statistical analyses were performed with SPSS 22 for Windows (SPSS Inc., Chicago, IL, USA). Categorical data are presented as frequencies and percentages. Chi-square analysis was conducted to test the demographic differences among people of different Sasang constitution types, depending on several variables (sex, age, BMI, and hemophilia type). Differences were considered significant at *P* < 0.05.

## 3. Results

### 3.1. Classification of Hemophilia Patients Based on General Characteristics

First, the 61 hemophilia patients were classified according to Sasang typology based on their general characteristics (Table 2). The patients were classified as Tae-Eum (60.7%), So-Yang (29.5%), and So-Eum (9.8%). The patients were evenly distributed across all age groups. The percentage of each Sasang type among hemophilia patients was different from 48.2%, 37.9%, 13.9% of the normal Korean population, respectively [18]. It indirectly suggests that Tae-Eum people have higher incidence of hemophilia, while So-Yang or So-Eum people have lower incidence. In addition, most of the hemophilia patients belong to type A (VIII). It tends to exhibit a higher incidence in patients of a particular Sasang type, Tae-Eum (49.2%) than So-Yang (16%) and So-Eum (9.8%). However, further studies are required to prove if an increased incidence of hemophilia is associated with a specific Sasang type. Although not very significant, it should be considered that Tae-Eum patients have much higher BMI than So-Yang and So-Eum patients.

### 3.2. Classification of Hemophilia Patients Based on Sasang Typology and Symptoms

As shown in Table 3, the 61 hemophilia patients were classified into four types according to Sasang typology. They consisted of Tae-Eum, 37 (60.7%); So-Yang, 18 (29.5%); and So-Eum, 6 (9.8%). When the patients were classified based on disease pattern or symptoms from the viewpoint of Sasang Constitutional Medicine, among the 37 Tae-Eum patients, 21 had Joyeol (dry-heat symptomatic pattern), 14 had Ganyeol (liver-heat symptomatic pattern), one had Janggam (prolonged infection pattern), and one had Wiwan-han (esophagus-cold symptomatic pattern). The 18 So-Yang patients consisted of six patients with the Yin-depletion symptomatic pattern (heat-related diarrhea accompanied by headache), eight patients with the Hunggeok-yeol symptomatic pattern (chest-heat symptomatic pattern), two patients with the Eumheo-oyeol symptomatic pattern, and two patients with the So-yang-sangpung symptomatic pattern. The six So-Eum patients included three patients with the Ulgwang symptomatic pattern.

### 3.3. General Disease of Hemophilia Patients Based on Sasang Typology

To determine whether a specific Sasang type was more prone to a particular disease, we investigated the general diseases of the patients (Table 4). Among the 37 Tae-Eum patients with hemophilia, five had diabetes (8.2%) and three had high blood pressure (4.9%). None of the 18 So-Yang patients or the six So-Eum patients had diabetes or high blood pressure.

## 4. Discussion

In this study, we analyzed Sasang typology as a preventive care strategy for hemophilia patients and investigated whether a specific Sasang type was associated with a higher incidence of a specific disease in hemophiliacs. We anticipated that the analysis of Sasang typology would help to prevent potential diseases that could occur among hemophiliacs and to provide patients with safe and effective care to help them avoid life-threatening complications. Finally, our study was intended to improve both the quality of life and the longevity of hemophilia patients with congenital bleeding disorders.

The distribution of Sasang types among hemophilia patients was not different from that of the normal Korean population. According to the Korea Constitutional Multicenter Bank (KCMB), which collected data from 3,711 study participants (1,353 men and 2,358 women) based on Sasang Constitutional Medicine (SCM) [19], the estimated constitutional distributions were as follows: 39.2% for Tae-Eum, 27.1% for So-Eum, 33.7% for So-Yang, and less than 0.1% for Tae-Yang. Most of the hemophilia patients were of the Tae-Eum type, and many of them had Joyeol (the dry-heat symptomatic pattern). However, a specific Sasang type did not seem to be associated with a higher incidence of a specific hemophilia type. Above all, the number of hemophilia patients with diabetes or high blood pressure was higher in the Tae-Eum type than in the other types, So-Yang and So-Eum. In previous studies among Koreans, each Sasang type exhibited a different susceptibility to specific disorders. In particular, the Tae-Eum type was found to have the highest prevalence of metabolic syndrome and its associated disorders. Irritable bowel syndrome (IBS) was most prevalent in So-Eum type [11]. The prevalence of insulin resistance (IR) also differed among the Sasang types, implying that the constitutional type of an individual can act as an independent risk factor for IR [20]. The prevalence of hypertension was also the highest in Tae-Eum [21].

Even morality and longevity were affected by an individual's Sasang types [4]. Sasang classification is based on benevolence-righteousness-propriety-wisdom, manifestations of sorrow-anger-joy-pleasure (Seong and Jeong). If it is considered that diseases can be induced by excessive sorrow and anger, the mind may greatly and unilaterally affect the body, resulting in illness. Thus, it is said that health originates from morality. Realizing and acting in accordance with the right moral behavior are essential for leading a healthy life. These concepts about disease are also emerging in western society. Prophylaxis is regarded as the optimal preventive care strategy, and increasing the quality of life has become the objective of care. Thus, it should follow that patients and their families recognize psychotherapy, physiotherapy, and community life as important for health [22].

In addition, medical improvements have allowed hemophilia patients to anticipate an increased quality of life and life expectancy. Hemophilia patients enjoy a life expectancy at birth that is close to that of males in the general population. Thus, increasing numbers of hemophilia patients are developing cardiovascular diseases and cancer in old age, presenting a new challenge to medical doctors and caregivers who treat or care for older people with hemophilia [23]. Proper physical exercise can provide hemophilia patients with benefits such as bleeding reduction in muscles and joints and can also reduce the incidence of metabolic and cardiovascular diseases and cancer in older people with hemophilia.

## 5. Conclusions

This study was designed to analyze potential diseases in hemophilia patients, as a preventive care strategy. From this study, it is concluded that hemophilia patients with Tae-Eum type should be treated through management according to SCM as well as with medicine against hemophilia as preventive care in order to avoid their pathological heat symptomatic pattern from being worsened pathologically. In addition, diabetes and high blood pressure should be regularly monitored in patients with Tae-Eum type. Although only 61 patients were recruited for this study we intend to recruit more patients in the future so that the potential diseases specific to hemophilia can be more precisely evaluated. Even though this study had the limitation of a small number of participating patients, to our knowledge, it was the first to investigate the potential diseases of hemophilia patients based on Sasang typology. Long-term preventive care for hemophilia patients with SCM will help to increase their quality of life and life expectancy.

## Figures and Tables

**Table 1:** Demographic data of hemophilia patients.

		*n* (%)
Sex	Male	59 (96.7)
	Female	2 (3.3)

Age	<30 years	23 (37.7)
	30–50 years	25 (41.0)
	>50 years	13 (21.3)

BMI		
≤18.5	Low weight	12 (19.7)
18.5–22.9	Normal weight	32 (52.5)
23.0–24.9	Overweight	15 (24.6)
≥25	Obesity	2 (3.3)

Hemophilia type	A (VIII)	52 (85.2)
	B (IX)	7 (11.5)
	C (VII)	1 (1.6)
	VWD	1 (1.6)

**Table 2:** General characteristics based on Sasang typology of Hemophilia Patients: chi test.

Sasang typology	*N* = 61	So-Yang	Tae-Eum	So-Eum	*χ* ^2^	*p*
Sex	Male	18 (30.2)	35 (55.6)	6 (9.5)	1.34	0.511
	Female	0 (0.0)	2 (4.8)	0 (0.0)		

Age	≤30 years	5 (9.4)	16 (25.0)	2 (3.1)	3.76	0.439
	30–50 years	8 (14.1)	13 (21.9)	4 (6.2)		
	≥50 years	5 (7.8)	8 (12.5)	0 (0.0)		

BMI						
≤18.5	Low weight	5 (8.2)	3 (4.9)	4 (6.6)	22.94	0.001
18.5–22.9	Normal weight	13 (22.6)	17 (29.0)	2 (3.2)		
23.0–24.9	Overweight	0 (0.0)	15 (24.2)	0 (0.0)		
≥25	Obesity	0 (0.0)	2 (1.6)	0 (0.0)		

Hemophilia type	A (VIII)	16 (26.2)	30 (49.2)	6 (9.8)	2.78	.882
	B (IX)	2 (3.3)	5 (8.2)	0 (0.0)		
	C (VII)	0 (0.0)	1 (1.6)	0 (0.0)		
	VWD	0 (0.0)	1 (1.6)	0 (0.0)		

**Table 3:** General symptoms according to Sasang typology of hemophilia patients.

	*n* (%)
Tae-Yang	0 (0.0)

So-Yang	18 (29.5)
Eumheo-oyeol	2 (11.1)
Soyan-sangpung	2 (11.1)
Yin-depletion	6 (33.3)
Hyunggyeok-yeol	8 (44.4)

Tae-eum	37 (60.7)
Joyeol	21 (56.8)
Wiwan-han	1 (2.7)
Ganyeol	14 (37.8)
Janggam	1 (2.7)

So-eum	6 (12.5)
Ulgwang	3 (50.0)
Tae-eum-obesity	1 (16.7)
Tae-eum-jaundice	1 (16.7)
Tae-eum	1 (16.7)

**Table 4:** General disease of hemophilia patients according to Sasang typology.

	So-Yang	Tae-Eum	So-Eum
Diabetes	0 (0.0)	5 (8.2)	0 (0.0)
High blood pressure	0 (0.0)	3 (4.9)	0 (0.0)
Hepatitis B	1 (1.6)	0 (0.0)	0 (0.0)
Hepatitis C	5 (8.2)	4 (6.6)	1 (1.6)
Stroke	1 (1.6)	0 (0.0)	0 (0.0)
Others	1 (1.6)	2 (3.3)	0 (0.0)
None	10 (16.4)	23 (37.7)	5 (8.2)

## Data Availability

The data used to support the findings of this study are included within the article.
